# Endoscopic retrograde cholangiopancreatography discharge tool combined with rapid trypsinogen-2 test to predict same-day discharge: a prospective cohort study

**DOI:** 10.1097/MEG.0000000000003014

**Published:** 2025-06-18

**Authors:** Christina J. Sperna Weiland, Megan M.L. Engels, Robbert C.H. Scheffer, Bas Van Balkom, Koen van Hee, Bertram J.T. Haarhuis, Joost P.H. Drenth, Jeanin E. van Hooft, Peter D. Siersema, Erwin J.M. van Geenen

**Affiliations:** a Department of Gastroenterology and Hepatology, Radboudumc, Nijmegen; b Department of Research and Development, St. Antonius Ziekenhuis, Nieuwegein; c Department of Gastroenterology and Hepatology, Leiden University Medical Center, Leiden; d Department of Gastroenterology and Hepatology, Jeroen Bosch Ziekenhuis, Den Bosch; e Department of Gastroenterology and Hepatology, Bernhoven, Uden; f Department of Gastroenterology and Hepatology, Amsterdam UMC, Amsterdam; g Department of Gastroenterology and Hepatology, Erasmus MC University Medical Center, Rotterdam, The Netherlands

**Keywords:** endoscopic retrograde cholangiopancreatography, patient discharge, pancreatitis, trypsinogen

## Abstract

**Objectives:**

Identifying patients at high-risk for endoscopic retrograde cholangiopancreatography (ERCP)-related adverse events (AEs) is important for postendoscopic discharge management. This study assesses two strategies, a urinary trypsinogen-2 (UT-2) dipstick combined with a risk-factor-based ERCP discharge tool, for identifying patients at increased risk of developing AEs.

**Methods:**

Between August 2018 and March 2021, 268 patients were enrolled in a multicenter prospective cohort. Sensitivity, specificity, positive predictive value (PPV), and negative predictive value (NPV) of the UT-2 dipstick, the discharge tool, and combined strategies were assessed for predicting ERCP-related AEs.

**Results:**

Twenty-four (10.5%) AEs occurred in the eligible 228 patients, of which 14 (6.1%) were post-ERCP pancreatitis. The discharge tool and UT-2 dipstick combination outperformed the individual strategies for all AEs with a sensitivity of 66.7% (95% CI, 44.7–84.4%), specificity of 78.5% (95% CI, 72.2–83.9%), PPV of 26.6% (95% CI, 19.8–34.8%) and NPV of 95.3% (95% CI, 91.9–97.3%). For post-ERCP pancreatitis alone, the strategies combined had a sensitivity of 64.3% (95% CI, 35.1–87.2%), specificity of 76.2% (95% CI, 69.9–81.7%), PPV of 14.9% (95% CI, 10.0–21.7%) and NPV of 97.0% (95% CI, 94.2–98.5%).

**Conclusion:**

Although the combination of UT-2 dipstick and discharge tool outperforms the two strategies separately in predicting post-ERCP AEs, we would not recommend implementation of either strategy given the low sensitivity when applied separately or combined.

## Background and aims

Endoscopic retrograde cholangiopancreatography (ERCP) has an approximate 10% risk of adverse events (AEs) [1,2], with post-ERCP observation policies varying between hospitals. Identifying patients requiring clinical observation who are at high-risk for ERCP-related AEs, including pancreatitis, is therefore essential for postendoscopic discharge management. The guideline of the European Society of Gastrointestinal Endoscopy (ESGE) suggests that patients with postprocedural abdominal pain should undergo serum amylase or lipase measurements 2–6 h after ERCP to determine whether same-day discharge is possible [3]. If the values are below the respective cutoff values of 1.5 and 4 times the upper limit of normal, patients can be discharged, as the risk of developing post-ERCP pancreatitis is considered negligible.

The discharge strategy proposed by the ESGE has several limitations. First, post-ERCP AEs such as infection, bleeding, and perforation are not accounted for. Second, post-ERCP hyperamylasemia is seen in up to 15% of asymptomatic patients [4]. Finally, the (logistical) burden of post-ERCP blood sampling and waiting for laboratory test results leads to a necessity for a postendoscopic short-stay unit, which incurs additional costs. These logistical issues prevent routine pancreatic enzyme testing in clinical practice, as demonstrated by a 2014 practice survey [5]. In an ideal setting, an easy-to-use (early) discharge tool would be simple, reliable, and cheap with a minimal burden to patient and hospital logistics.

Two alternatives for post-ERCP adverse event prediction are a risk-factor-based discharge tool and the rapid measurement of urinary trypsinogen-2 (UT-2) [6,7]. The discharge tool incorporates a multi-item scoring prognostic model which differentiates between patients with a high adverse event risk (27%) and those with a low to moderate adverse event risk (8%). However, this tool has not been validated externally (Supplementary information, Supplemental digital content, https://links.lww.com/EJGH/B178) [6]. The urinary dipstick is based on an immunofluorometric assay with a cutoff value of 50 μg/l trypsinogen-2 [7]. UT-2 is accurate in diagnosing post-ERCP pancreatitis, with a sensitivity of 86% and specificity of 94% [8,9].

The aim of the current study was to assess the performance of the ERCP discharge tool, the UT-2 dipstick, and a combination of these strategies as an alternative to the discharge strategy proposed by the ESGE guidelines. By combining both strategies, we hypothesize that post-ERCP adverse event prediction could be more accurate than the UT-2 dipstick and discharge tool separately.

## Materials and methods

This multicenter prospective cohort study was conducted in accordance with the Declaration of Helsinki. The Medical Ethical Review Committee of the coordinating academic hospital approved this study and waived the need for written informed consent (Approval number: 2018-4431). Verbal consent was deemed sufficient given the only interventional aspect from the patients’ perspective was the urinary dipstick test. The study was internally funded by a quality improvement grant. Reporting was performed in line with the Standards for Reporting Diagnostic Accuracy Studies (STARD) statement [10].

### Participants and study procedure

Patients were enrolled in one academic hospital (Radboudumc, Nijmegen, the Netherlands) and two community hospitals (Bernhoven, Uden, the Netherlands; Jeroen Bosch Ziekenhuis, Den Bosch, the Netherlands) between August 2018 and March 2021. Patients over 18 years undergoing an ERCP with informed consent for study participation were included in a convenience series. Prior sphincterotomy was not considered grounds for exclusion. Ongoing acute pancreatitis was the sole exclusion criterion. The ERCP was performed by eight endoscopists with advanced endoscopy training (Radboudumc: two; Bernhoven: two; and Jeroen Bosch Ziekenhuis; four) according to professional standards. Just before the start of the ERCP, all patients received 100 mg rectal nonsteroidal anti-inflammatory drugs (NSAIDs), consisting of diclofenac or indomethacin, unless contraindicated due to allergy or renal insufficiency. Pancreatic duct (PD) stents for the prevention of post-ERCP pancreatitis were placed at the discretion of the treating endoscopist. All patients were admitted for one night to monitor the development of post-ERCP AEs. Patients were followed up by a telephone inquiry 5 days after ERCP to evaluate symptoms related to post-ERCP AEs.

### Definitions

The start time of ERCP was defined as scope-to-mouth contact. Post-ERCP pancreatitis was diagnosed according to the Cotton criteria [11]. The severity of post-ERCP pancreatitis was classified according to the revised Atlanta criteria [12] and Cotton criteria [11]. Other ERCP-related AEs (e.g. perforation, bleeding, and infection) were also classified by the Cotton criteria [11]. Cases with uncertainty regarding the occurrence of an adverse event were discussed with a blinded expert endoscopist (E.J.M.v.G.).

### Discharge strategies

The discharge tool includes a prognostic model with patient- and procedure-related risk factors [6]. Patients were scored two points if the underlying disease indication was primary sclerosing cholangitis and one point for (precut) sphincterotomy, suspicion of sphincter of Oddi dysfunction (SOD), younger age <60 years, female, history of pancreatitis, pancreas divisum, or difficult cannulation (defined as >10 min). A score of three or larger placed the patient in the observation group. As described in detail in the original publication [6], these risk factors for post-ERCP AEs were based on a literature review and multivariate analysis on a retrospective cohort (*n* = 588). Subsequent validation in a prospective cohort of 220 patients distinguished between 27% AEs in the high-risk group and 8% risk in the low- to intermediate-risk group. This validated prognostic model was incorporated in a discharge tool, adding other patient- and procedure-related risk factors for postprocedural perforation and bleeding to distinguish which patients are eligible for early discharge after ERCP (Supplementary Figure 1, Supplemental digital content, https://links.lww.com/EJGH/B178).

The UT-2 dipstick test (Actim Pancreatitis, MedixBiochemica, Kauniainen, Finland) was performed directly from voided urine collected in a cup by the treating nurse. The instructions were to aim to take the samples 2 hours after initiation of ERCP; procedure duration and first postprocedural urine voiding could cause exact time variation. The 2-hour timeframe was based on the maximum observation time possible in the postendoscopy recovery room as discussed with the participating hospitals and the minimum expected time needed for UT-2 levels to rise sufficiently [13]. The UT-2 dipstick had a cutoff value of 50 μg/l and a positive test colored two clear blue lines within 5 min. A negative test was defined as one blue line and an ‘invalid’ test was defined as the absence of both blue lines. In case of a first invalid test result, a second UT-2 dipstick was used in the same urine sample. If this was subsequently also invalid, the test was definitively considered invalid. If the result of the UT-2 dipstick in a patient was positive, this patient was placed in the one-night observation group as defined by the discharge tool for the analysis.

### Outcomes

The primary outcome of the study was the diagnostic accuracy of the individual and combined discharge strategies (discharge tool and UT-2) in predicting post-ERCP AEs, calculated as sensitivity, specificity, positive predictive value (PPV), and negative predictive value (NPV). The secondary outcome of the study was the diagnostic accuracy of the strategies for post-ERCP pancreatitis only.

### Data collection

Data was collected prospectively using standardized data collection forms for the endoscopist and the nurse who performed the UT-2 dipstick. The advanced endoscopist reported details of the procedure including ERCP procedure time, the use of post-ERCP pancreatitis prophylaxis, cannulation attempts and duration, and (inadvertent) PD cannulation. If the standardized data collection form was incomplete regarding cannulation attempts, the patient was excluded from analyses. The nurse registered the collection time and result of the dipstick on a separate case report form. If the nurse failed to record the result of the UT-2 dipstick on the standardized data collection form, the test was considered ‘missing’. Patients with ‘invalid’ or ‘missing’ UT-2 dipstick results were excluded from analyses. Collected data was verified by two researchers (C.J.S.W. and M.M.L.E.) through patient chart review. All study authors had access to the study data and approved the final manuscript.

### Sample size calculation

The sample size of 226 ERCPs was calculated based on an 80% likelihood of detecting, with a 5% significance level, an increase in sensitivity from 91% in the use of the discharge tool alone to 99% in combination with the UT-2 dipstick test. Considering a 15% dropout rate, the sample size estimate was 260 patients.

### Statistical analysis

Baseline and ERCP characteristics that were dichotomous or categorical were stated as absolute numbers and percentages of the total. Continuous values were stated as means with SD or medians with interquartile range (IQR). For the primary analysis, the sensitivity, specificity, PPV, and NPV of both the UT-2 dipstick and discharge tool were calculated with a 95% confidence interval (CI) for all ERCP-related AEs and separately for post-ERCP pancreatitis.

## Results

**Fig. 1.**
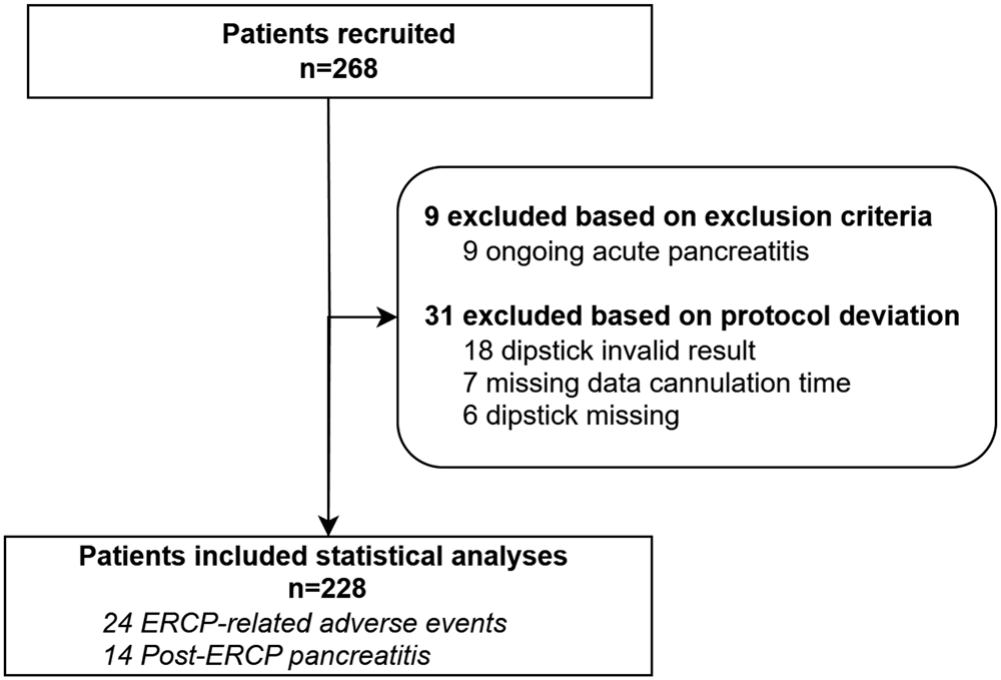
Flowchart inclusion and exclusion. ERCP, endoscopic retrograde cholangiopancreatography; PEP, post-ERCP pancreatitis.

A total of 268 patients who underwent ERCP were enrolled in three participating centers between August 2018 and March 2021 (Fig. 1). Nine patients were excluded due to ongoing acute pancreatitis at the time of ERCP. Thirty-one additional patients with missing data from the discharge tool or a ‘missing’ or ‘invalid’ UT-2 dipstick were excluded from further analyses (*n* = 228).

### Baseline and endoscopic retrograde cholangiopancreatography characteristics

The median age of the 228 included patients was 68.6 years (IQR, 54.9–77.1) with 95 male patients (41.7%)(Table 1). Choledocholithiasis was the suspected underlying disease in 147 (64.5%) patients and cancer in 42 (18.2%) patients. The proportion of other underlying diseases can be found in Supplementary Appendix Table S1, Supplemental digital content, https://links.lww.com/EJGH/B178. In some patients, multiple underlying diseases were present or suspected. Median ERCP duration was 23.0 min (IQR, 14.5–37.0). Cannulation was achieved in 226 patients (99.1%), with a prolonged cannulation time (>10 min) in 27 (11.8%) procedures. (Table 2). Guidewire cannulation of the PD occurred in 76 (33.3%) and contrast injection into the PD in 40 (17.5%) patients. PD stent placement was successful in seven out of eight attempts. Fifteen patients (6.6%) did not receive rectal NSAID prophylaxis due to renal insufficiency (*n* = 7), reported allergy (*n* = 5) or for unknown reasons (*n* = 3). In total, 14 (6.1%) patients developed post-ERCP pancreatitis, two (0.9%) bleeding, three (1.3%) perforation, and seven (3.1%) cholangitis. New onset of upper abdominal pain at 24 hours after ERCP was documented in 28 patients of which 13 were diagnosed with post-ERCP pancreatitis (one with a concurrent perforation), one patient with a perforation and bleeding, and four patients developed cholangitis. More detailed information regarding the population who developed post-ERCP pancreatitis can be found in Supplementary Appendix Table S2, Supplemental digital content, https://links.lww.com/EJGH/B178.

**Table 1.** Baseline characteristics

Baseline characteristics	*n* = 228^a^
Birth sex	
Male, *n* (%)	95 (41.7)
Age, median (IQR)	68.6 (54.9–77.1)
BMI, median (IQR) *n* = 225	25.4 (23.2–29.0)
Medical history and comorbidity, *n* (%)	
Cholecystectomy	65 (28.5)
Acute pancreatitis	26 (11.4)
Pancreatic cancer	25 (11.0)
Recurrent acute pancreatitis	11 (4.8)
Chronic pancreatitis	10 (4.4)
Post-ERCP pancreatitis	7 (3.1)
Pancreatic surgery	3 (1.3)
Altered anatomy^b^	3 (1.3)
(Suspicion of) SOD	3 (1.3)
Biliary carcinoma	3 (1.3)
ASA classification, *n* (%)	
I	21 (9.2)
II	113 (49.6)
III	94 (41.2)
IV	0

ASA, American Society of Anesthesiologists Physical Status; ERCP, endoscopic retrograde cholangiopancreatography; IQR, interquartile range; SOD, Sphincter of Oddi Dysfunction.

a Percentages are given as a valid percentage of patients whose data was complete for a certain variable.

b Surgically altered anatomy complicating the ERCP procedure. Defined as anatomical variations in which bile and/or pancreatic secretions (in case of pancreatic duct interventions) do not enter the duodenum by way of the ampulla of Vater.

**Table 2.** ERCP characteristics and outcomes

ERCP characteristics and outcomes	*n* = 228^a^
ERCP duration, min, median (IQR) *n* = 217	23.0 (14.5–37.0)
Sedation, *n* (%)	
Midazolam	42 (18.4)
Propofol	183 (80.3)
General anesthesia	3 (1.3)
Complexity,^b^ *n* (%)	
1	26 (11.4)
2	168 (73.7)
3	32 (14.0)
4	2 (0.9)
Trainee involvement,^c^ *n* (%)	43 (18.9)
ERCP before for same indication, *n* (%)	40 (17.5)
Naïve papilla (no prior cannulation), *n* (%)	164 (71.9)
Cannulation duration, *n* (%)	
≤10 min	201 (88.2)
>10 min	27 (11.8)
PD guidewire passage, *n* (%)	76 (33.3)
PD contrast injection, *n* (%)	40 (17.5)
Bleeding during ERCP, *n* (%)	2 (0.9)
No NSAID prophylaxis, *n* (%)	15 (6.6)
(Attempted) PD stent placement, *n* (%)	8 (4.1)
Post-ERCP pancreatitis	14 (6.1)
Mild^d^	1/11
Moderate^d^	10/2
Severe^d^	2/0
Missing severity	1
Post-ERCP bleeding	2 (0.9)
Mild	0
Moderate	2
Severe	0
Post-ERCP perforation	3 (1.3)
Mild	0
Moderate	1
Severe	2
Post-ERCP cholangitis	7 (3.1)
Mild	2
Moderate	5
Severe	0

ERCP, endoscopic retrograde cholangiopancreatography; IQR, interquartile range; NSAID, nonsteroidal anti-inflammatory drugs; PD, pancreatic duct.

a Percentages are given as a valid percentage of patients whose data was complete for the certain variable.

b American Society for Gastrointestinal Endoscopy (ASGE) grading scale [14].

c Defined as at least one cannulation attempt by the trainee.

d First number according to Cotton *et al*. [13] Second number according to revised Atlanta criteria [12].

### Discharge tool

The discharge tool predicted that 43 patients needed observation, of whom 10 developed an AE (23.3%) (Supplementary Table S3, Supplemental digital content, https://links.lww.com/EJGH/B178). In the discharge group, there was a 7.6% adverse event rate (14/185). Sensitivity, specificity, PPV, and NPV for the discharge tool for all AEs were therefore 41.7% (95% CI, 22.1–63.4), 83.8% (95% CI, 78.0–88.6), 23.2% (95% CI, 14.6–34.8), and 92.5% (95% CI, 89.7–94.5), respectively.

Post-ERCP pancreatitis was present in five patients of the predicted observation group (11.6%) versus nine patients in the predicted discharge group (4.9%). The performance for the discharge tool in detecting post-ERCP pancreatitis is shown in Table 3.

**Table 3.** Performance (with 95% CI) of discharge tool, UT-2 dipstick, and combined strategies

	Sensitivity (95% CI)	Specificity (95% CI)	PPV (95% CI)	NPV (95% CI)
All adverse events *n* = 24				
Discharge tool	41.7% (22.1–63.4)	83.8% (78.0–88.6)	23.2% (14.6–34.8)	92.5% (89.7–94.5)
UT-2 dipstick	33.3% (15.6–55.3)	95.1% (91.2–97.6)	44.4% (25.9–64.6)	92.4% (90.2–94.2)
Combination	66.7% (44.7–84.4)	78.5% (72.2–83.9)	26.6% (19.8–34.8)	95.3% (91.9–97.3)
Post-ERCP pancreatitis *n* = 14				
Discharge tool	35.7% (12.8–64.9)	82.2% (76.5–87.1)	11.6% (5.8–21.8)	95.2% (93.0–96.7)
UT-2 dipstick	42.9% (17.7–71.1)	94.4% (90.4–97.1)	33.2% (18.0–52.9)	96.2% (94.2–97.6)
Combination	64.3% (35.1–87.2)	76.2% (69.9–81.7)	14.9% (10.0–21.7)	97.0% (94.2–98.5)

CI, confidence interval; ERCP: endoscopic retrograde cholangiopancreatography; NPV, negative predictive value; PPV, positive predictive value; UT-2, urinary trypsinogen-2.

### UT-2 dipstick

The dipstick test was performed at a median of 2 hours 45 min (IQR, 2:07 - 4:18) after the start of the ERCP in the 211 patients in whom timing was available. A positive test result of the dipstick was found in 18 patients, of whom six developed post-ERCP pancreatitis. Eight patients with post-ERCP pancreatitis were missed with the UT-2 dipstick. The dipstick had a sensitivity of 42.9% (95% CI, 17.7–71.1), specificity of 94.4% (95% CI, 90.4–97.1), PPV of 33.2% (95% CI, 18.0–52.9), and an NPV of 96.2% (95% CI, 94.2–97.6) for predicting post-ERCP pancreatitis (Table 3 and Supplementary Table S3, Supplemental digital content, https://links.lww.com/EJGH/B178). If patients with a positive dipstick are placed in the observation group and patients with a negative dipstick discharged, the observation group would have had a 33% (6/18) risk of developing post-ERCP pancreatitis and the discharged patients a risk of 3.8% (8/210).

### Combination of discharge tool and UT-2 dipstick

**Fig. 2.**
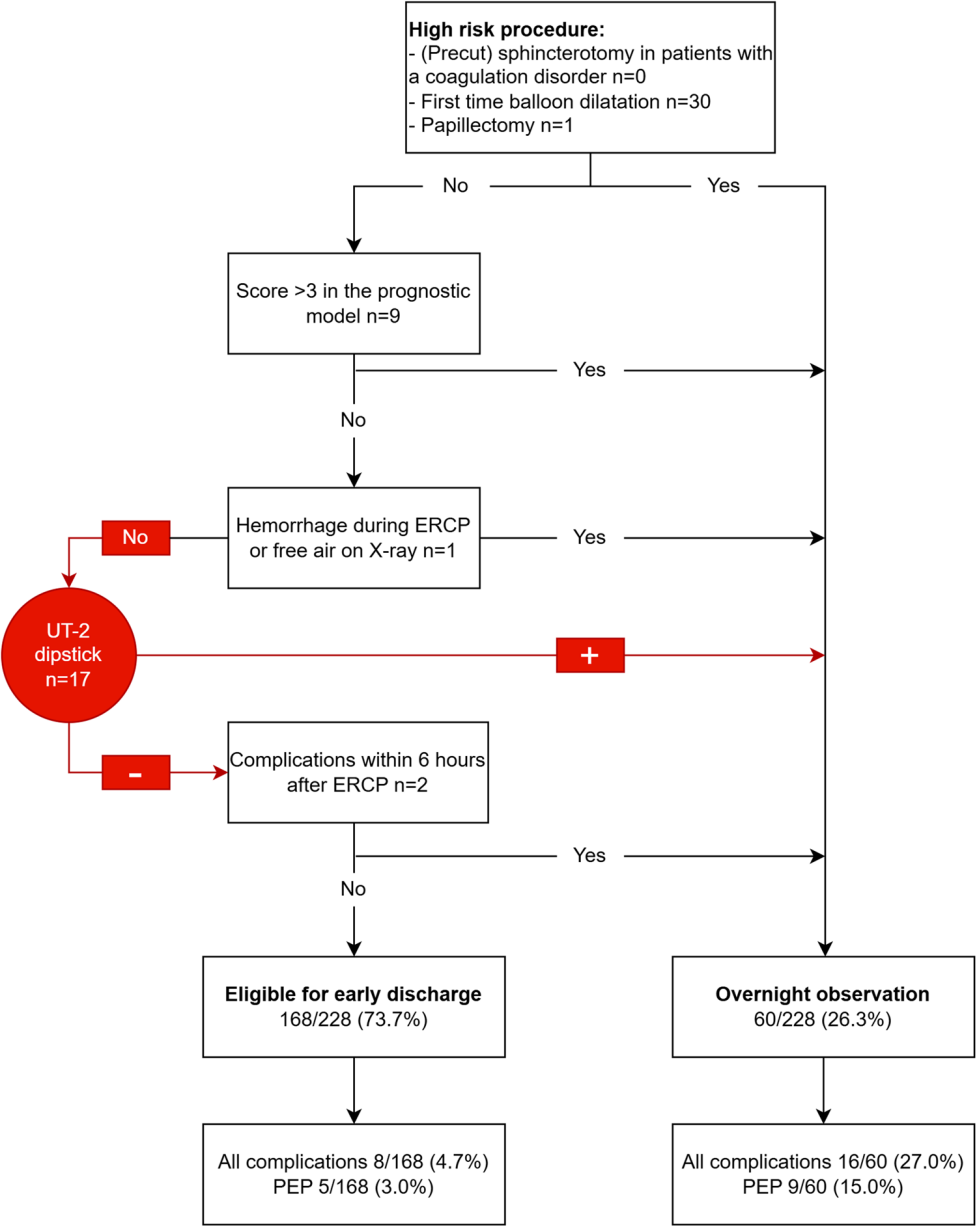
UT-2 dipstick implemented in the discharge tool adapted from Jeurnink *et al*. [6]. ERCP, endoscopic retrograde cholangiopancreatography; PEP, post-ERCP pancreatitis; UT-2, urinary trypsinogen-2.

The discharge tool and UT-2 dipstick results were incorporated as demonstrated in Figure 2. For all AEs, the observation group had a 27.0% (16/60) AE rate and the discharge group had a 4.7% (8/168) AE rate. The combination of strategies had a sensitivity of 66.7% (95% CI, 44.7–84.4), specificity of 78.5% (95% CI, 72.2–83.9), PPV of 26.6% (95% CI, 19.8–34.8), and NPV of 95.3% (95% CI, 91.9–97.3) for all AEs (Table 3 and Supplementary Table S3, Supplemental digital content, https://links.lww.com/EJGH/B178). Combined use of both the discharge tool and dipstick was able to differentiate between a 15.0% (9/60) post-ERCP pancreatitis risk in the observation group and a 3.0% (5/68) post-ERCP pancreatitis risk in the discharge group. The performance of the combination in detecting post-ERCP pancreatitis is shown in Table 3.

### Post hoc analysis UT-2 dipstick excluding gastrointestinal malignancies

Given that UT-2 has been shown to be elevated in the absence of pancreatitis and the presence of gastrointestinal tumors, we performed a post hoc analysis excluding all gastrointestinal cancers (*n* = 41). A cohort of 187 patients remained in which AEs occurred in 17 patients including 11 cases of post-ERCP pancreatitis. By excluding gastrointestinal malignancies, the standalone UT-2 test achieved a higher specificity (97.7 vs. 94.4%) and PPV (50.0 vs. 33.2%), similar NPV (96.1 vs. 96.2%), and lower sensitivity (36.4 vs. 42.9%) for post-ERCP pancreatitis detection. Supplementary Appendix Table S4, Supplemental digital content, https://links.lww.com/EJGH/B178 contains the results for all adverse events, the discharge tool, and the combination of discharge strategies in this subgroup.

## Discussion

In this prospective multicenter study, the combination of the UT-2 dipstick and discharge tool was superior to both strategies alone in predicting safe early discharge after ERCP. Applied to the clinical setting, 74% of patients would be eligible for early discharge with a low-risk of ERCP-related AEs (NPV > 95%). However, the sensitivity of both strategies was markedly lower than shown in previous studies [6,9,13,15,16] and raises doubts regarding its suitability for clinical implementation.

The discharge tool was able to differentiate between a high- (23%) and low-intermediate- (8%) risk of developing ERCP-related AEs in our study. The combination with the UT-2 dipstick showed a slightly improved differentiating capacity between a high (27%) and low to moderate (5%) risk of developing ERCP-related AEs. It is conceivable that risk factors may have changed since the development of the discharge tool due to the current use of routine preventive measures for post-ERCP pancreatitis, such as rectal NSAIDs and intensive intravenous hydration [17,18]. However, robust literature is missing on the contribution of individual risk factors in the development of post-ERCP pancreatitis in a patient receiving prophylaxis. Current guidelines do not provide a clear definition of low- or high-risk patients, mentioning mainly risk factors from studies predating routine prophylaxis use [3]. Hence, we opted not to stratify results based on perceived post-ERCP pancreatitis risk due to the lack of consensus on the precise criteria for defining a high-risk patient.

The dipstick test for post-ERCP pancreatitis detects trypsinogen-2, which is also secreted by biliary endothelia and released during nonspecific pancreatic injury. It has been shown to be elevated in the presence of gastrointestinal tumors [15,16,19–21]. In our cohort, a gastrointestinal malignancy was the indication for ERCP in 8/12 positive dipsticks in the absence of post-ERCP pancreatitis (cholangiocarcinoma *n* = 6, pancreatic adenocarcinoma *n* = 1, and ampullary adenocarcinoma *n* = 1). Excluding patients with gastrointestinal tumors from a discharge decision based on the UT-2 dipstick test led to a higher test specificity and PPV in our post hoc analysis by reducing the number of false positives, at the expense of introducing selection bias to the use of the UT-2 dipstick. Additionally, consensus on the optimal timing of UT-2 measurement is lacking [9,13,15,16,22,23], with earlier studies stating 1–6 hours [13,15,16], and a recent study reporting best performance at 24 hours [9]. We adhered to 2 hours after the start of ERCP, in line with several other recent publications [22,23], as this would allow patients to be discharged without admission at a short-stay unit. However, considering the low UT-2 dipstick sensitivity in our study, 2 hours might have been too early for UT-2 levels to increase sufficiently.

Recently, it has been reported that the UT-2 dipstick combined with abdominal pain had a sensitivity of 60% and an NPV of 98% for predicting post-ERCP pancreatitis at 4 hours post-ERCP [9]. If abdominal pain was not included, the performance of the dipstick dropped and was similar to our combined strategies with sensitivity, specificity, PPV, and NPV of 60%, 63%, 6%, and 98%, respectively. There is a large variance in UT-2 dipstick performance published, as illustrated by the most recent publications on this topic. Obaitan *et al*. [22] reported 11% sensitivity (nine post-ERCP pancreatitis cases in 254 patients) which contrasts with Hama *et al*. [23] reporting a 100% sensitivity (five post-ERCP pancreatitis cases in 100 patients). Another surprising finding was the 30% baseline UT-2 test positivity in the Rainio *et al*. study [9]. This proportion of pre-ERCP positive tests is concerning, and the lack of baseline UT-2 measurements in our study design can therefore be considered a limitation. It is worth noting that baseline test positivity was lower in other recent studies (2–8%) and predominantly affected the specificity and PPV [22,23].

We assessed the performance of two different ERCP discharge strategies and their combination in a prospective multicenter setting with few exclusion criteria. Comparable AEs and successful cannulation rates ensure our results are representative for clinical practice and can likely be extrapolated to other populations undergoing ERCP. Although our study was performed in a large prospective cohort of patients undergoing ERCP for various indications, there are some limitations. Serum pancreatic enzyme measurements were not part of the study protocol and were only performed if ERCP-related AEs were suspected. As previously stated, serum amylase and lipase measurement are more invasive compared to the point-of-care urinary dipstick test, and the laboratory turnaround time is a hindrance in early discharge, which restricts its use in clinical practice.

This study addresses a clinically relevant for early discharge after ERCP. We demonstrated that by applying the combined strategies approach 67% of AEs that occurred can be detected while also reducing overnight hospital stays by 74%. Given the variable UT-2 dipstick performance in previous studies and low sensitivity in our study, we acknowledge that current data is insufficient to recommend clinical implementation. Future research should focus on redefining risk factors in the era of improved prophylaxis, by computer modeling in adequately powered prospective studies [24]. A comparison of the UT-2 dipstick with 2–4 hours of serum amylase/lipase levels in selected patients who develop abdominal pain after an ERCP, the recommended target population for these tests by the ESGE [3], is another topic worth considering.

In conclusion, the combination of UT-2 dipstick and discharge tool outperforms the two strategies separately, with a 27% adverse event risk in the observation group and 5% in the early discharge group. Nevertheless, the current performance does not sufficiently support widescale clinical implementation, and future research toward other optimal ERCP discharge strategies is required.

## Acknowledgements

The study was internally funded by a quality improvement grant from the Radboudumc.

The study was registered with Dutch Trial Register: NL8486.

All authors contributed to the study's conception and design. Material preparation, data collection, and analysis were performed by C.J.S.W. and M.M.L.E. The first draft of the manuscript was written by C.J.S.W. and M.M.L.E, and all authors commented on previous versions of the manuscript. All authors read and approved the final manuscript.

### Conflicts of interest

There are no conflicts of interest.

## Supplementary Material


