# Supplementary material for: Endoscopic retrograde cholangiopancreatography discharge tool combined with rapid trypsinogen-2 test to predict same-day discharge: a prospective cohort study
Source: Eur J Gastroenterol Hepatol. 2025 Jun 18;37(11):1206–12. doi: 10.1097/MEG.0000000000003014 (PMC12462681; doi:10.1097/MEG.0000000000003014)
Supplement: Supplementary file 1 [file ejgh-37-1206-s001.docx]

**Supplementary Information**

This appendix has been provided by the authors to give readers additional information about their work.

**ERCP discharge tool combined with rapid trypsinogen-2 test to predict same-day discharge - *a prospective cohort study***

C.J. Sperna Weiland*, M.M.L. Engels*, R.C.H. Scheffer, B. Van Balkom, K. van Hee, B. Haarhuis, J.P.H. Drenth, J.E. van Hooft, P.D. Siersema, E.J.M. van Geenen

**Supplementary figure 1**. Discharge tool as proposed by Jeurnink et al. (*Springer Nature Switzerland AG*) (6)


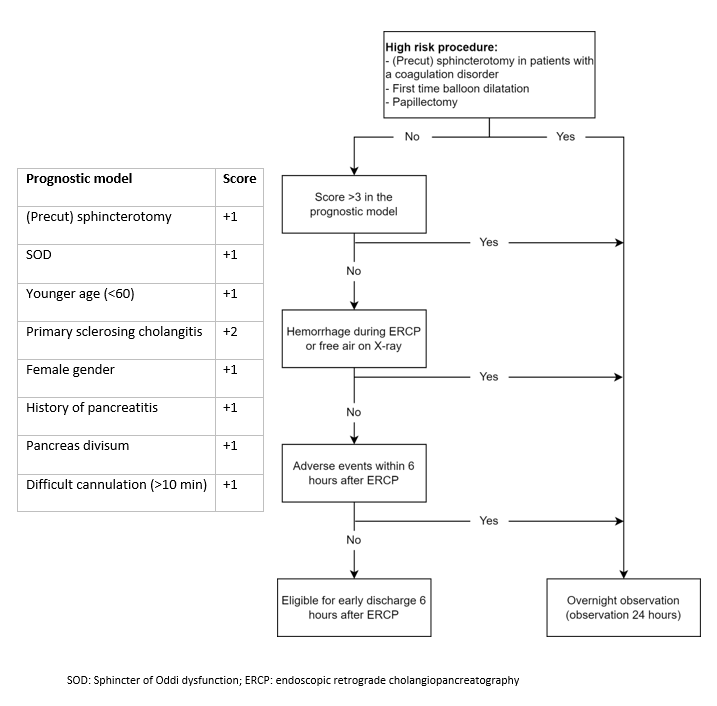


SOD: Sphincter of Oddi dysfunction; ERCP: endoscopic retrograde cholangiopancreatography

**Table S1**. Suspected underlying disease

| (Suspected) underlying disease^a^ – no. (%) | n=228 |
| --- | --- |
| Choledocholithiasis/sludge | 147 (64.5) |
| Pancreatic adenocarcinoma | 24 (10.5) |
| Cholangitis | 15 (6.6) |
| Benign stricture bile duct | 11 (4.8) |
| Cholangiocarcinoma | 11 (4.8) |
| Chronic pancreatitis | 6 (2.6) |
| Metastatic cancer | 6 (2.6) |
| (Postoperative) bile leak | 5 (2.2) |
| Pancreatic stricture unknown origin | 2 (0.9) |
| Primary sclerosing cholangitis | 2 (0.9) |
| Sphincter of Oddi dysfunction | 2 (0.9) |
| Acute pancreatitis | 1 (0.4) |
| Pancreas divisum | 1 (0.4) |
| Ampullary adenoma | 1 (0.4) |
| Ampullary adenomacarcinoma | 1 (0.4) |
| Internally migrated biliary stent | 1 (0.4) |
| ^a^ It is possible patients were categorized as having two underlying diseases, therefore total = 236 | |

**Table S2**. Detailed information of post-ERCP pancreatitis cases

| **Post-ERCP pancreatitis** | **n=14** |
| --- | --- |
| No rectal NSAIDs no. (%) | 2 (14.2) |
| Due to allergy | 1 |
| Due to renal insufficiency | 1 |
| PD guidewire passage – no. (%) | 7 (50.0) |
| PD contrast injection – no. (%) | 4 (28.6) |
| ERCP: endoscopic retrograde cholangiopancreatography, PD: pancreatic duct | |

**Table S3.** Contingency tables for Discharge tool, UT-2 dipstick and combination of tools

1. **Discharge tool**

|  | ERCP-related comp + | ERCP-related comp - | Total |
| --- | --- | --- | --- |
| Discharge tool + | 10 | 33 | 43 |
| Discharge tool - | 14 | 171 | 185 |
| Total | 24 | 204 | 228 |
| ERCP: endoscopic retrograde cholangiopancreatography | | |  |

|  | PEP + | PEP- | Total |
| --- | --- | --- | --- |
| Discharge tool+ | 5 | 38 | 42 |
| Discharge tool- | 9 | 176 | 185 |
| Total | 14 | 214 | 228 |
| PEP: post-ERCP pancreatitis | |  |  |

1. **UT-2 dipstick tool**

|  | ERCP-related comp + | ERCP-related comp - | Total |
| --- | --- | --- | --- |
| UT-2 + | 8 | 10 | 18 |
| UT-2 - | 16 | 194 | 210 |
| Total | 24 | 204 | 228 |
| UT-2: urinary trypsinogen-2; ERCP: endoscopic retrograde cholangiopancreatography | | | |

|  | PEP + | PEP- | Total |
| --- | --- | --- | --- |
| UT-2 + | 6 | 12 | 18 |
| UT-2 - | 8 | 202 | 210 |
| Total | 14 | 214 | 228 |
| UT-2: urinary trypsinogen-2; PEP: post-ERCP pancreatitis | | |  |

1. **Combination UT-2 dipstick and Discharge tool**

|  | ERCP-related comp + | ERCP-related comp - | Total |
| --- | --- | --- | --- |
| Combination + | 16 | 44 | 60 |
| Combination - | 8 | 160 | 168 |
| Total | 24 | 204 | 228 |
| ERCP: endoscopic retrograde cholangiopancreatography | | | |

|  | PEP + | PEP- | Total |
| --- | --- | --- | --- |
| Combination + | 9 | 51 | 60 |
| Combination - | 5 | 163 | 168 |
| Total | 14 | 214 | 228 |
| PEP: post-ERCP pancreatitis | |  |  |

Table S4. Post-hoc analysis excluding malignancies (n=187)

|  | Sensitivity (95% CI) | Specificity (95% CI) | PPV  (95% CI) | NPV (95% CI) |
| --- | --- | --- | --- | --- |
| All adverse events n=17 | | | | |
| Discharge tool | 41.2%  (18.4-67.1) | 81.2%  (74.5-86.8) | 18.0%  (10.3-29.5) | 93.2%  (90.2-95.4) |
| UT-2 dipstick | 23.5%  (6.8-49.9) | 97.7%  (94.1-99.4) | 50.0%  (21.5-78.5) | 92.7%  (90.7-94.33) |
| Combination | 58.8%  (32.9-81.6) | 78.3%  (71.3-84.2) | 21.3%  (14.2-30.6) | 95.0%  (91.5-97.1) |
| Post-ERCP pancreatitis n=11 | | | | |
| Discharge tool | 36.4%  (10.9-69.2) | 80.1%  (73.4-85.7) | 10.3%  (4.7-20.9) | 95.3%  (92.8-96.9) |
| UT-2 dipstick | 36.4%  (10.9-69.2) | 97.7%  (94.3-99.4) | 50.0%  (22.4-77.6) | 96.1%  (94.0-97.5) |
| Combination | 63.6%  (30.8-89.1) | 77.3%  (70.4-83.2) | 14.9%  (9.4-22.8) | 97.1%  (93.9-98.7) |
| CI: confidence interval; PPV: positive predictive value; NPV: negative predictive value; ERCP: endoscopic retrograde cholangiopancreatography; UT-2: urinary trypsinogen-2 | | | | |
